# Leser–Trélat sign associated with lung cancer: a case report and review of the literature

**DOI:** 10.3389/fonc.2026.1995820

**Published:** 2026-10-29

**Authors:** Mei Xiong, Lengping Chen, Junwu Zhou

**Affiliations:** Department of Dermatology, The First People’s Hospital of Shuangliu District, West China (Airport) Hospital of Sichuan University, Chengdu, China

**Keywords:** case report, eruptive seborrheic keratoses, Leser–Trélat sign, lung adenocarcinoma, paraneoplastic dermatoses, visceral malignancy

## Abstract

The Leser–Trélat sign is a rare paraneoplastic dermatosis characterized by the abrupt development of multiple seborrheic keratoses within a short period and its association with visceral malignancies. Although gastrointestinal adenocarcinoma is the most commonly reported associated malignancy, lung cancer has increasingly been recognized as a possible underlying tumor. We report a case of a 62-year-old man who developed eruptive seborrheic keratoses on the neck, anterior chest, and back in conjunction with the diagnosis of minimally invasive adenocarcinoma of the right upper lobe. We additionally reviewed eight previously reported cases of lung cancer-associated Leser–Trélat sign and summarized their clinical characteristics. Notably, the paraneoplastic manifestation developed at an exceptionally early tumor stage (T1a(mi)N0Mx), suggesting that the Leser–Trélat sign may occur even in minimally invasive pulmonary adenocarcinoma. Adenocarcinoma was the predominant histological subtype among these cases (6 of 9), further suggesting a potential association between the Leser–Trélat sign and adenocarcinoma. Clinicians should be aware of this cutaneous marker, as its recognition may contribute to the early detection of occult malignancies.

## Introduction

The Leser–Trélat sign is a rare paraneoplastic dermatosis characterized by the abrupt onset of multiple seborrheic keratoses with rapid increases in number and size, and is associated with underlying visceral malignancies (1). The condition was named after the nineteenth-century surgeons Edmund Leser and Ulysse Trélat, who independently reported cutaneous lesions in patients with malignancy; however, the lesions they initially described were likely cherry angiomas rather than seborrheic keratoses. It was not until the early 20th century that Hollander described the association between eruptive seborrheic keratoses and visceral malignancy, after which the eponymous term became widely adopted.

The Leser–Trélat sign has been most frequently reported in association with adenocarcinomas, particularly gastrointestinal adenocarcinomas (including gastric, colorectal, and rectal cancers), as well as lymphoproliferative neoplasms, breast cancer, and lung cancer (1). Although the underlying pathogenesis remains unclear, circulating tumor-derived growth factors, including transforming growth factor-α (TGF-α) and epidermal growth factor (EGF), have been proposed as potential mediators (2). The sign may precede, coincide with, or follow the diagnosis of malignancy, and partial or complete regression of the cutaneous lesions after successful treatment of the primary tumor has been reported in approximately 50% of cases (3). Herein, we report a case of Leser–Trélat sign associated with minimally invasive pulmonary adenocarcinoma and review previously reported cases in the literature.

## Case description

A 62-year-old man presented with a 3-year history of brown maculopapules on the neck and a 1-year history of the sudden development and rapid enlargement of similar lesions on the anterior chest and back, accompanied by mild, intermittent pruritus. Dermatological examination revealed approximately 100 brown to dark-brown maculopapules, measuring 0.5–2.0 cm in diameter, on the neck, anterior chest, and back. The lesions were well demarcated, with rough, verrucous surfaces and a characteristic “stuck-on” appearance. No similar lesions were observed on the extremities or face, and there were no clinical features of acanthosis nigricans, palmoplantar hyperkeratosis, or skin fragility (**Figure 1**). Histopathological examination of an excised specimen from the largest lesion on the back revealed hyperkeratosis, acanthosis, and papillomatosis with pseudocomedone formation, consistent with seborrheic keratosis (**Figure 2**).

**Figure 1:**
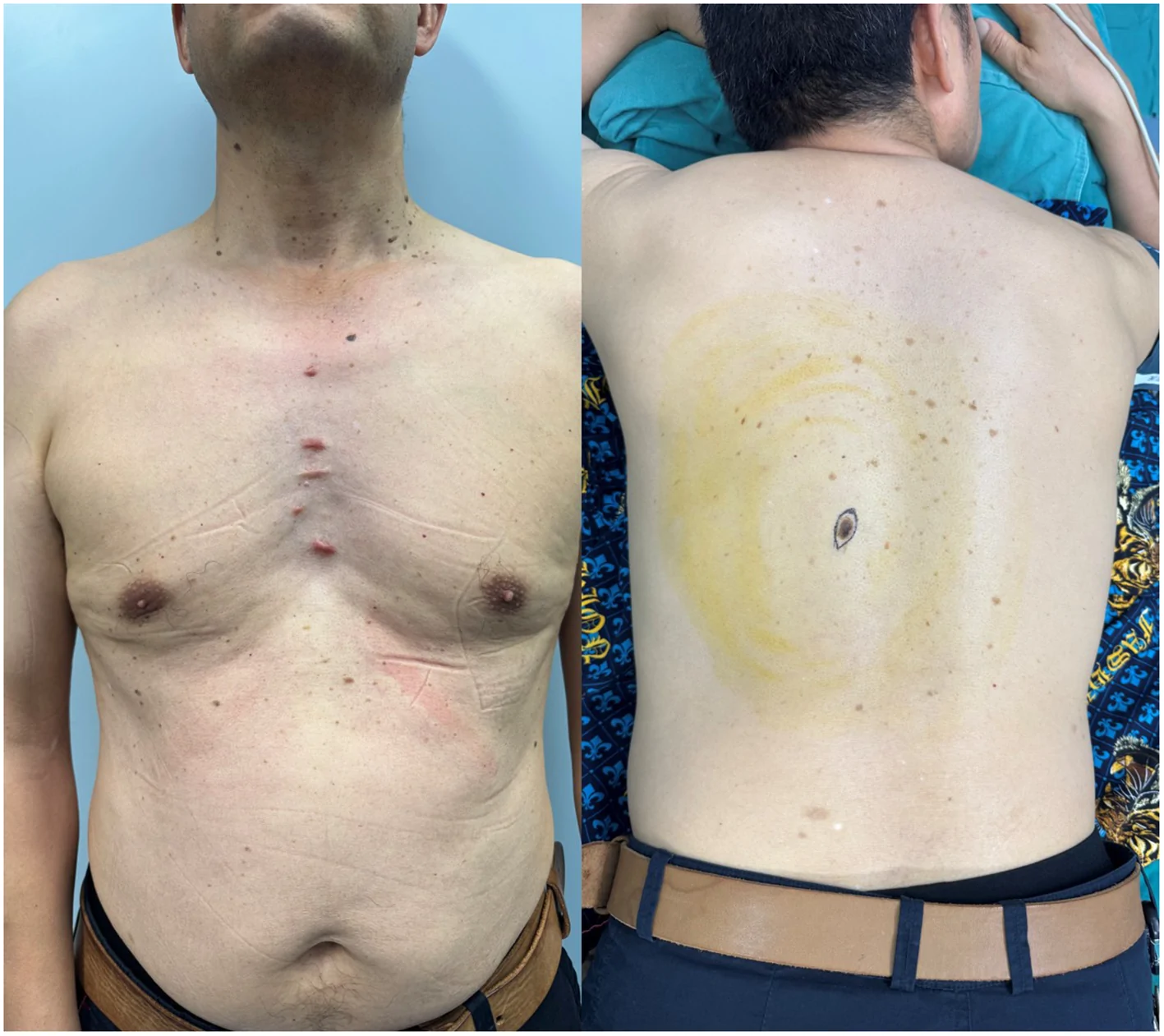
Scattered brown to dark-brown maculopapules with well-demarcated borders and rough, verrucous surfaces were present on the neck, anterior chest, and back.

**Figure 2:**
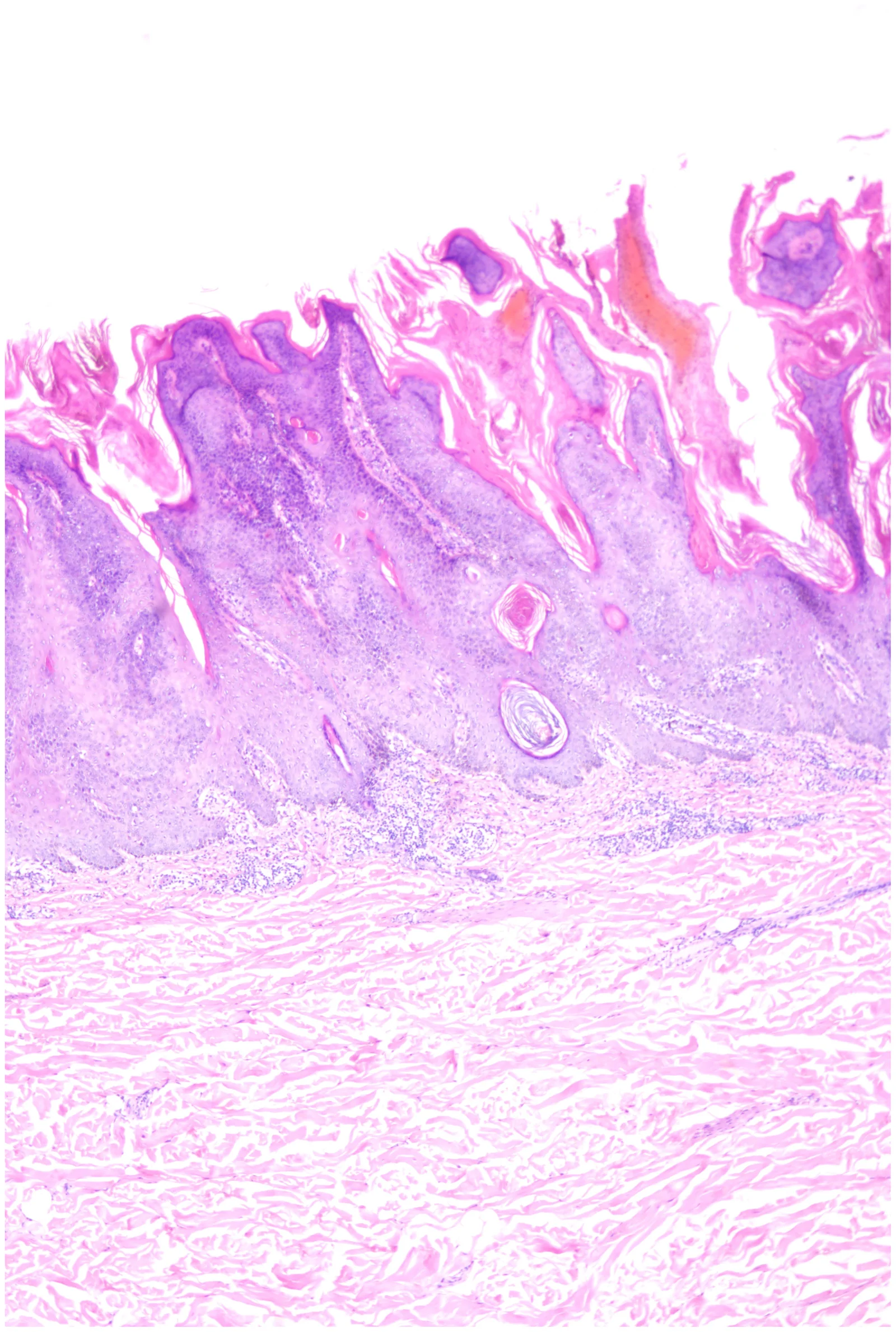
Histopathological examination of the lesion on the back revealed hyperkeratosis, acanthosis, and papillomatosis with pseudocomedone formation (HE ×10).

Given the eruptive nature of the lesions, the patient’s medical history was reviewed for potential underlying systemic disease. The patient reported that chest computed tomography (CT) performed during a routine health examination 1 year earlier had revealed a patchy area of increased density in the right upper lobe. The lesion was subsequently resected and histopathologically diagnosed as minimally invasive adenocarcinoma, measuring 1.2 × 0.9 × 0.6 cm (TNM stage T1a(mi)N0Mx). Following pulmonary resection, the cutaneous lesions showed no further increase in number or size during follow-up, although no spontaneous regression was observed. In addition, hematologic tests and gastrointestinal endoscopy were performed, and CT of the chest, abdomen, and brain showed no evidence of malignancy.

## Discussion

We report a case of Leser–Trélat sign in a 62-year-old man with minimally invasive pulmonary adenocarcinoma. To our knowledge, this is one of the few reported cases of Leser–Trélat sign presenting at such an early tumor stage (T1a(mi)N0Mx), suggesting that paraneoplastic cutaneous manifestations may occur even in the setting of early-stage, potentially curable malignancy.

We reviewed the English-language literature and identified eight previously reported cases of Leser–Trélat sign associated with lung cancer. Together with the present case, a total of nine patients were included in the analysis, with one publication reporting two patients (**Table 1**). The patients ranged in age from 58 to 85 years, consistent with the predominance of the sign among older adults (4). No clear sex predominance was observed (five men and four women). Regarding histological subtypes, six cases (66.7%) were adenocarcinoma, two were squamous cell carcinoma, and one was adenosquamous carcinoma, supporting the previously reported predominance of adenocarcinoma among associated malignancies (5). In most cases, the cutaneous lesions developed concurrently with or before the diagnosis of lung cancer. Notably, in three patients, the malignancy was detected through further evaluation prompted by the cutaneous manifestations.

**Table 1:** Summary of the clinical characteristics of reported cases of Leser–Trélat sign associated with lung cancer.

Study (first author, year)	Age (years)/Sex	Histological type	Timing of Leser–Trélat sign relative to tumor diagnosis
Mathien A, 2023 (8)	58/F	Lung adenocarcinoma (EGFR T790M mutation)	Concurrent
Khemakhem R, 2022 (12)	78/M	Adenocarcinoma (EGFR mutation-negative)	Prior
Khemakhem R, 2022 (12)	76/M	Lung adenosquamous carcinoma	After
Asri H, 2018 (13)	70/M	Lung adenocarcinoma	Prior
Sardon C, 2017 (14)	85/F	Lung squamous cell carcinoma	Prior
Jepsen RK, 2014 (7)	69/F	Lung adenocarcinoma (increased EGFR protein expression without mutation)	Concurrent
Ceylan C, 2002 (15)	74/M	Lung squamous cell carcinoma	Concurrent
Heaphy MR Jr, 2000 (6)	58/F	Lung adenocarcinoma	Prior
Present case	62/M	Minimally invasive adenocarcinoma	Concurrent

EGFR, epidermal growth factor receptor; F, female; M, male. “Prior” indicates that the cutaneous lesions developed before the diagnosis of the tumor; “Concurrent” indicates that the cutaneous lesions developed concurrently with the diagnosis of the tumor; “After” indicates that the cutaneous lesions developed after the diagnosis of the tumor.

Several hypotheses have been proposed to explain the pathogenesis of the Leser–Trélat sign. The most widely accepted growth factor hypothesis proposes that tumor-derived humoral factors may play a central role. Heaphy et al. first proposed that tumor-secreted factors, such as TGF-α, may contribute to the acute eruption of seborrheic keratoses and the concurrent development of acanthosis nigricans (6). Elevated levels of EGF and insulin-like growth factor have also been reported in some patients (2). Studies of the epidermal growth factor receptor (EGFR) pathway have provided further insights into the potential mechanisms involved. Jepsen et al. reported high levels of EGFR protein expression in tumor cells from patients with lung adenocarcinoma and malignant pleural mesothelioma associated with the sign and proposed that tumor-associated EGFR ligands or an EGFR autocrine loop might contribute to the development of seborrheic keratoses (7). Further support for a potential role of EGFR signaling comes from a report of Leser–Trélat sign in a patient with primary lung adenocarcinoma harboring an EGFR T790M mutation (8). Nevertheless, the precise pathogenic mechanisms underlying the Leser–Trélat sign remain to be elucidated.

The association between eruptive seborrheic keratoses and visceral malignancy remains controversial. Some authors have questioned whether this association reflects a true causal relationship or merely a coincidence, given that seborrheic keratoses are extremely common among older adults, who are also at increased risk of malignancy (9). The term “pseudo-Leser–Trélat sign” has been proposed to describe eruptive seborrheic keratoses in the absence of an underlying malignancy (10). Nevertheless, numerous case reports, particularly those involving younger patients in whom seborrheic keratoses are otherwise uncommon, have supported the possibility that the sign represents a genuine paraneoplastic phenomenon (11). A further terminological debate concerns whether the terms “sign” or “syndrome” should be used when an underlying malignancy is identified. In this report, we consistently use “sign” in accordance with prevailing clinical usage.

The present case highlights several clinically relevant points. First, the Leser–Trélat sign may be associated with lung adenocarcinoma at an exceptionally early stage (T1a(mi)N0Mx), indicating that the cutaneous manifestation may precede the clinical recognition of a small, potentially curable tumor. Second, the occurrence of eruptive seborrheic keratoses in temporal association with the diagnosis of lung cancer highlights the potential value of evaluating patients with eruptive lesions for occult malignancy. Third, adenocarcinoma was the predominant histological subtype among the lung cancer-associated cases (66.7%), in keeping with the predominance of adenocarcinoma reported in the broader literature.

Several limitations should be acknowledged. As a case report with a literature review, the small number of included cases limits the generalizability of the findings. The retrospective nature of the literature review and reliance on published case reports may have introduced selection, reporting, and publication biases. In addition, EGFR mutation testing was not performed in our patient, limiting our ability to explore the potential molecular mechanisms underlying the association. The CT images from the earlier examination were also unavailable, so the radiological evolution of the pulmonary lesion could not be illustrated. Finally, the relatively short follow-up period limits our ability to assess the long-term clinical course of the cutaneous lesions.

## Conclusions

In conclusion, we report a case of Leser–Trélat sign in a 62-year-old man with minimally invasive pulmonary adenocarcinoma and identified eight additional cases through a review of the literature. Adenocarcinoma was the predominant histological subtype among these lung cancer-associated cases, suggesting a possible association between Leser–Trélat sign and pulmonary adenocarcinoma. The development of eruptive seborrheic keratoses, particularly when they arise abruptly or increase rapidly in number or size, should prompt consideration of an underlying malignancy and further evaluation when clinically appropriate. Recognition of this potential cutaneous marker may facilitate the detection of malignancy at an early, potentially curable stage.

## Data Availability

The original contributions presented in the study are included in the article/supplementary material. Further inquiries can be directed to the corresponding author.
